# A qualitative study examining the sustainability of shared care in the delivery of palliative care services in the community

**DOI:** 10.1186/1472-684X-12-32

**Published:** 2013-08-29

**Authors:** Lily DeMiglio, Allison M Williams

**Affiliations:** 1 School of Geography and Earth Sciences, McMaster University, 1280 Main Street West, Hamilton, Ontario L8S 4K1, Canada

**Keywords:** Shared care model, Palliative care services, Qualitative research, Scale, Community

## Abstract

**Background:**

This paper focuses on the sustainability of existing palliative care teams that provide home-based care in a shared care model. For the purposes of this study, following Evashwick and Ory (2003), sustainability is understood and approached as the ability to continue the program over time. Understanding factors that influence the sustainability of teams and ways to mitigate these factors is paramount to improving the longevity and quality of service delivery models of this kind.

**Methods:**

Using qualitative data collected in interviews, the aim of this study is twofold: (1) to explore the factors that affect the sustainability of the teams at three different scales, and; (2) based on the results of this study, to propose a set of recommendations that will contribute to the sustainability of PC teams.

**Results:**

Sustainability was conceptualized from two angles: internal and external. An overview of external sustainability was provided and the merging of data from all participant groups showed that the sustainability of teams was largely dependent on actors and organizations at the local (community), regional (Local Health Integration Network or LHIN) and provincial scales. The three scales are not self-contained or singular entities but rather are connected. Integration and collaboration within and between scales is necessary, as community capacity will inevitably reach its threshold without support of the province, which provides funding to the LHIN. While the community continues to advocate for the teams, in the long-term, they will need additional supports from the LHIN and province. The province has the authority and capacity to engrain its support for teams through a formal strategy. The recommendations are presented based on scale to better illustrate how actors and organizations could move forward.

**Conclusions:**

This study may inform program and policy specific to strategic ways to improve the provision of team-based palliative home care using a shared care model, while simultaneously providing direction for team-based program delivery and sustainability for other jurisdictions.

## Background

A product of health care restructuring that has taken place across the globe is regionalization [1,2]. In Ontario, this form of restructuring involved the implementation of 14 Local Health Integration Networks (LHINs), which are geographically bound health planning regions in the mid-2000s, to improve the integration of health services and to include local citizens in health care decisions. Alongside the LHINs, End-of-Life Networks (now referred to as Hospice Palliative Care Networks), were implemented in order to improve palliative care (PC) in each LHIN [3]. The Hospice PC Networks are groups of stakeholders that identify local priorities and the appropriate service delivery models for their jurisdictions. The service delivery model endorsed in the LHIN of concern in this study was the implementation of enhanced PC teams that act as experts to support primary care providers, such as community nurses that are put in place through the service coordinating agency, the Community Care Access Centre (CCAC), and family physicians, using a shared care model.

Due to both the shift of care and the shift in place of death from institutions such as hospitals, to community-based settings [4], more patients are receiving end-of-life care at home. In addition, patients often prefer to die at home [5]. While there has been a shift from hospital to community care for the dying, the provision of care in the home by family physicians has not followed suit. According to a Canadian study by Brenneis et al. [6], family physicians are more willing to provide community-based care if they are supported through changes to fee schedules and access to consultants, remedial education and home care services for their patients. Along the same lines, Australian-based research by Yuen and colleagues [7] also suggests that the ability to keep patients at home requires commitment from family physicians to do home visits with the support of an enhanced specialist team.

In order to better support family physicians and other health care providers in the community, many diverse community-based models and initiatives have developed across Canada eg. [8-11]. As noted above, the community-based model endorsed by the Hospice PC Network in the LHIN of concern in this study involved the implementation of enhanced PC teams that act as experts to support primary care providers. At the time of the study, five teams were in place and serving five of the 11 delineated communities in the LHIN area. The overall intended goal was to introduce teams in the remaining communities as resources became available. At the end of data collection, one additional team had formed.

This paper focuses on existing PC teams that provide home-based care in a shared care model. Drawing on the work of Moorehead [12] and Chomik [13], Howell et al. [14] describe the heterogeneous nature of shared care models: “shared care models may differ in their structure and composition but share a common goal of mobilizing the skills and knowledge of a range of health professionals, including medical specialists, in the planned delivery and joint responsibility for a patient population” (p. 61). The teams included in this study vary in terms of their structure but, at a minimum, consist of a physician, nurse and social worker with expertise and/or advanced training in PC. Shared care is established when the team works in consultation with family physicians and community nurses. In our previous research with the teams of concern herein, identified barriers, such as lack of funding for non-physician team members and the inability to secure buy-in from primary care providers, were shown to have posed challenges in their pursuit of the shared care model [15]. Additional research has also explored the factors or facilitators employed by the teams to overcome challenges, such as securing funding for non-physician team members, and undertaking capacity building initiatives with primary care providers [16]. Using qualitative data collected in interviews, the aim of this study is twofold: (1) to explore the factors that affect the sustainability of the teams, and; (2) based on the results of this study, propose recommendations that will contribute to the sustainability of PC teams. For the purposes of this study, following Evashwick and Ory [17], sustainability is understood and approached as the ability to continue the program over time. Understanding factors that influence the sustainability of teams and ways to mitigate these factors is paramount to improving the longevity and quality of service delivery models of this kind. This study may inform program and policy specific to strategic ways to improve the provision of team-based palliative home care using a shared care model, while simultaneously providing direction for team-based program delivery and sustainability. Following a description of the methods, research findings are presented followed by a discussion and concluding remarks and recommendations.

## Methods

This paper is based on the results of a longitudinal case study of five PC teams that work in consult in a shared care model to provide care to patients in the home setting in Ontario, Canada. The data were collected from September 2010 to September 2011, and, as common in case study research, data were collected from multiple sources [18]; here, semi-structured qualitative interviews were conducted with a variety of individuals including PC team members, key-informants and stakeholders. A detailed description of the study design has been reported elsewhere [16].

Ethics approval from McMaster University was obtained in advance of data collection. All participants provided written and informed consent prior to being interviewed. Due to the tight-knit nature of the PC community in the study site, specific geographic details are not provided to ensure confidentiality.

### Participants

All participants were involved in either the direct (e.g., practitioners) or indirect delivery of PC services (e.g., researchers, program administrators), with the exception of one key-informant who had expertise in the mental health shared care model. A purposive sampling strategy was used to recruit a rich sample of participants [19] and to enhance credibility [20]. Each of the five PC teams, representing a combination of rural and urban jurisdictions (i.e. two rural, 2 small urban & 1 large urban), was invited to participate in a series of three focus group interviews (i.e., spaced four months apart). The first team was formed in 1996/1997, and the remaining teams were formed between 2001 and 2010. Most of the teams were based at a hospice or hospital and only one team was based in a service coordinating agency. If medical and/or nursing learners were present during the time of interview, they were given the option to participate. Since each team varied in terms of composition, the average number of focus group participants also varied between teams. On average two of the teams had three participants, while the remaining teams averaged between five to eleven participants per focus group session. Six key-informants who were knowledgeable in the area of shared care models through research, clinical practice or program planning, agreed to participate in one-on-one interviews. Lastly, seven stakeholders were asked to participate in one-on-one interviews based on their vested interest in the PC teams through their role in either the management and/or delivery of PC programs in various sectors such as hospice, hospital and service agencies.

### Data collection

With permission from the participants, all interviews were digitally recorded. Separate interview schedules for the PC teams, key-informants and stakeholders were developed by the researchers, guided by both the literature and the research objectives. Table 1 provides an abbreviated list of the types of questions asked during the focus group sessions and one-on-one interviews. Teams were interviewed at their home base location while key-informants and stakeholders were interviewed at a location of their choosing. All of the interviews were conducted by one of the researchers (LD).

**Table 1:** Focus group and one-on-one interview questions

**Interview participant(s)**	**Questions**
**PC Teams**	When did the team form?
	Has the membership changed since the team formed? If yes, please outline any changes.
	How long did it take for the team to start working in the community once it formed? (Follow-up: Was it a short or lengthy process? Why? Is this process ongoing? Why?]
	What has impacted the speed of working in the community?
	In which settings does your team work? (E.g. hospital, long-term care, home, hospice)
	What do you feel has facilitated the functioning of the team to date?
	What barriers have presented themselves in the successful functioning of the team?
	Do you feel the community is aware of your team’s services?
	Does geography impact your team? (Probe: in terms of collaboration, urban centre versus rural centre)
	What are some of the barriers/facilitators that your team experiences in providing care?
	If you could give one piece of advice to a team that is just starting out, what would it be?
	In terms of your team’s sustainability, and when I use the term sustainability I mean your team’s capacity to work together over time and to continue doing what you’re doing, what factors contribute to your team’s sustainability? (Probe: funding, collaboration, cooperation of stakeholders, policies etc.)
	Are there factors that hinder your team’s sustainability? (Probe: stress, burnout, funding, politics etc.)
	What do you foresee as the most probable challenge your team will have to face in the next five years? Ten years?
	From a policy perspective, at a local level, are there any new policies or modifications to existing policies that would better support teams? At a LHIN level? At a provincial level?
	Do you think a shared care service model like yours should be consistent across the province? Why? Why not?
**Key informants**	What is your understanding of shared care model(s)?
	In your opinion, what are the advantages/disadvantages to providing PC using a shared care model?
	In your opinion what are the barriers/facilitators to providing PC using a shared care model? (Probe: system/institution, policies, clinician attitudes etc.)
	What are the necessary minimum conditions needed to form a PC team?
	In your opinion, how important is geography to PC teams? (Probe: being located in the same workspace, having a home-base, the geography served by the team etc.)
	How is success measured with respect to: (i) team development and; (ii) team sustainability?
**Stakeholders**	What is your understanding of shared care models?
	Are you familiar with the PC team and the shared care model that is used in the X LHIN area?
	Do you think that this model of care is working in this LHIN area? Why? Why not?
	What are your thoughts on using a shared care model to provide PC in the community? In hospice? In long-term care? In hospital?
	What are some of the barriers/facilitators to providing shared care? (Probe: system/institution, policies, clinician attitudes etc.)
	Can you discuss any changes that would help to improve/support community-based PC teams working in a shared care model? (Probe: team composition, policies, communication with partners, geography, resources etc.)
	Do you think that PC teams working in a shared care model are sustainable? Do you think these teams will be around in the next two to five years?
	What advice would you offer for implementing new community-based PC teams?

### Data analysis

The interviews were transcribed verbatim. The researcher (LD) examined each of the transcripts thoroughly through multiple readings. During the readings, recurring themes were documented in an iterative process. Similar to Giesbrecht et al.’s [21] study, participants repeatedly used prioritized scalar categories to articulate their responses. In particular, in discussing factors affecting the sustainability of teams, participants commonly used the terms “community”, “LHIN” and “province” as points of reference. As a result, thematic coding [22] involved the grouping of interview data according to these three common scalar categories.

The final step of the analysis involved merging the two participant datasets (team members and key-informants/stakeholders), which in turn bolstered the study’s validity through multiple triangulation (i.e., data and methodological triangulation) [23]. The information from key-informants, stakeholders, and team members were combined and areas of convergence eg. [24] around the factors that affect the sustainability of teams were explored.

## Results and discussion

The analysis of the focus group interview transcripts revealed that teams conceptualized sustainability in two different, yet interconnected, dimensions, referred to here as internal and external sustainability; this paper will focus primarily on external sustainability as team characteristics that contributed to internal sustainability were previously explored (see [16]). In what follows, external sustainability is explored in relation to scale.

It is useful to conceptualize the sustainability of the PC teams with the three scales of community, LHIN and province as the individual pillars that support the ongoing work of the teams. Based on this conceptualization, the foundation is the impetus to provide PC to patients at home (e.g., more demand, the emphasis on care in the community, population aging). The need for PC has the potential to grow and this corresponds to the width of each pillar which depicts : (1) the geographical area/size of the scale and; (2) the power/authority at each scale. As a result, here the understanding of scale aligns with Howitt’s [25] conceptualization of scale as size, level and relation, with the province being the largest and most powerful followed by the LHIN and finally, the community. Howitt [25] distinguished three elements of scale: (1) size (e.g., spatial, population); (2) level (e.g., hierarchies) and; (3) relation (e.g., culture, economy), arguing that scale should also be considered from a relational perspective because focusing on size and level alone would lead to discrepancies since scale is “better understood dialectically than hierarchically” (p. 52). There is strong support for not viewing scale as a vertical hierarchy eg. [1,26], as this can be construed as disempowering the local in comparison to the global. External sustainability is now discussed from the perspective of team members (TM), key-informants (KI) and stakeholders (S), followed by scalar results. Quotes from participants are included as a means of providing context.

### External sustainability

The analysis revealed that although the teams operate at the community scale, their sustainability is affected by contextual issues, individual actors, partners and policies at the community, LHIN and provincial scales. As stated by Paasi [27]: “scales are also historically contingent; they are produced, exist and may be destroyed or transformed in social and political practices and struggles” (p. 542). Many, including Brenner [28], have also noted the political implications of scale and the relationship between geographic scale and politics. Brenner [28] argues that the politics of scale be examined with a plural rather than a singular focus, whereby the interrelationships amongst a range of geographic scales are examined. Along the same lines, Smith [29] explains the notion of “scale jumping” whereby “political claims and power established at one geographical scale are expanded to another” (p. 726). Given the conceptualization presented above,, the sustainability of the teams would be compromised without adequate support from all three of the scales (i.e. pillars of support). Although it would be possible for one of the pillars to provide more support than another at any given time, it would be unrealistic and problematic for the team to rely on just one or two of the three pillars. A team member explained that without the macro-scale support, the teams remained vulnerable:

“Like what continuity can we look toward for ourselves as a little entity, you know, in the big picture…” (TM)

Another team member elaborated on the role of decision-makers and managers in relation to their sustainability. Many team members attributed their continuity to the support of local managers and partners, who have witnessed their growth and success over time. Yet teams were aware that their futures often relied on the advocacy of certain individuals and, as a result, they recognized their vulnerability:

“…by the graces of people who’ve been around long enough, like there are enough folks who support us financially, who’ve been around to see the genesis of the team and the team’s success that they are supportive but managers come and go…” (TM)

The above excerpt demonstrates how external players (i.e., outside the team) have contributed to the continuity of the teams. The merging of data from team members, key-informants and stakeholders further categorizes external factors by Howitt’s [25] classification of scale, from the smallest, least powerful scale through to the largest, most powerful: (1) community; (2) LHIN and; (3) province, as will now be explored.

#### 1. Community

Each team provides services to patients in a geographically defined community. In effect, the five teams provide services to five communities that vary according to both physical and social environmental characteristics, such as population density and socio-demographics. The teams interact with a number of individual actors at this scale, including community nurses and family physicians, in addition to partners such as nursing agencies, acute care hospitals, long-term care facilities and residential hospices. According to participants, the sustainability of the teams is dependent on building collaborative partnerships with community actors and partners. Unlike other settings of PC, such as acute care hospitals, hospices and long-term care facilities, the community is not a contained place. As such, the data pointed to a broader and more extensive network of partners that needed to be invited to work in collaboration with the teams. To ensure an environment of collaboration, the data suggested that the team be autonomous or self-directed (e.g., via team consensual decision making) and, while team members should be accountable to their funders, teams should not be “owned” or micromanaged by their funders.

The formation of collaborative partnerships depends on the size of the community. Key-informants and stakeholders discussed that the size of the community will dictate the number of partners involved and the power of the partners involved (e.g., a community hospital in a rural community as opposed to a hospital corporation in an urban centre). Further, the size of the community may be more conducive to collaboration and relationship building, such that in smaller communities there are less providers and settings of care which, in turn, increases a team’s visibility:

“Potentially more rural or smaller communities are more able to know all the actors like all the family physicians know each other and the PC specialists can get together… the bigger you get, the more difficult it is to have that [sense of] community and therefore, I think in more urban settings you will see more [of a] substitution model….” (KI 1)

Also, in terms of community size, there are often fewer options in smaller communities. Clinicians are sometimes forced to work together because there are no other people to refer to whereas the pool of human health resources in urban communities tends to be larger.

Participants also agreed that individual actors and partners would be more willing to collaborate and work in shared care with teams if there were an after-hours on-call system. Only two of the five (rural and small urban) teams in the study had such a system. It was suggested that the five teams consider partnering amongst themselves as a means to provide an on-call system and to share resources when team members are absent due to vacation or otherwise. It was noted that additional funding would be required if such an on-call system were to be put into place. Such a system would enable teams to better support family physicians and to address the context in which many family physicians practice today:

*“… and the medical field has gotten away from home visiting like sort of in the*[26]*80s and*[26]*90s that was sort of country bumpkin doctors, so if you’re an urban doctor and you’re modern and hip, everything is in your office …”(S2)*

Another important area that fosters collaboration between providers is community rounds, which provide the opportunity for team members to build relationships with those outside of the team. Rounds enable teams to meet with primary care providers to discuss patients who are being cared for in the home setting. These meetings allow teams to use their expertise to build the PC capacity of primary care providers. According to a key-informant, community rounds also present the opportunity for interprofessional learning which benefits the patient through triaging to avoid crisis management.

Teams also discussed the need to train medical, nursing and social work learners about PC and interprofessional practice. They were concerned about the lack of interest in PC among young health care professionals. While the teams worked to mentor learners in the community, this could also be addressed at the provincial scale through medical education sectors and professional regulatory bodies.

The sustainability of teams in the community is largely based on their ability to build capacity and relationships with primary care providers, and to engage community partners in the shared care service delivery model. Teams also discussed the importance of building relationships with community stakeholders to raise awareness and gain support:

“I think we would want to foster a better understanding of our stakeholders and community care and their responsibility with respect to the community piece and pave the way for better communication across care settings and reciprocity across care settings and not feel like we exist out here without no one else accountable…” (TM)

Team members stressed that building relationships would in turn influence their workload in terms of issues related to the manageability of their caseload. They explained that earlier referrals and an on-call system would help to avoid crises, which, in turn would decrease their frustrations and provide greater job satisfaction.

While teams work at the community scale to enhance collaboration and partnerships, system-redesign at both the LHIN and provincial scale is required because community initiatives alone will not sustain the teams:

“…it’s on the will of the people and… the organizations to play fast and loose with the rules, be flexible with the money and say, ‘Okay I can protect this little piece for you, I can make that happen, we’ll pay for the parking, we’ll pay for the mileage’… but I mean I’ve seen things fall apart on mileage, because someone was covering the mileage and [then] they could no longer do it…” (S2)

The excerpt above illustrates that teams have benefited from innovative strategies and community support but even so, the lack of secure funding from the LHIN and provincial scales makes them vulnerable entities. In what follows, the factors affecting sustainability at the LHIN scale are discussed.

#### 2. LHIN

While the teams provide services to communities within the LHIN area, they are affected by the decisions and funding from the LHIN, which is the administrative body that manages, funds and coordinates all health services within a bounded geography. Teams must also work together with the CCAC, which is funded by the LHIN to coordinate home health care services for patients (e.g., community nurses and personal support workers). Case managers from the CCAC are considered as team members by three of the five teams, one of which is housed in a CCAC. There is also a Hospice PC Network for the LHIN which initially endorsed the teams and the shared care model for the LHIN area. At the time of study, the Hospice PC Network was in a period of transition, due to changes in leadership.

Key-informants and stakeholders discussed the importance of the home care service infrastructure. In order for teams to sustain the shared care model, and to support patients, home care services were paramount, both in terms of their availability and in the provision of skilled providers to deliver the service. The state of the community care infrastructure is largely dependent on funding from the provincial government. However, it was agreed that funding for the teams - and especially non-physician team members - from the LHIN, would ensure the long-term sustainability of the teams. It was and continues to be necessary for the LHIN to recognize that the teams and the shared care model are a worthy investment. At the time of the study, a neighbouring LHIN had received provincial support for a similar service model:

“… I think it’s a great example of when the LHIN is recognizing that it’s a worthwhile model, that it gives the results that they’re looking for and makes a commitment to it and says to the other partners – ‘This is important, you need to all work together’…” (KI 4)

This suggests that advocacy for and championing of the shared care teams from the LHIN is vital. Additionally, direction from the Hospice PC Network in terms of planning and execution was also discussed. Participants were impressed with the Hospice PC Network’s efforts in ensuring that teams were housed in the best possible environment, allowing them to have meeting places that were conducive for clinical learning and confidential conversations. The data also determined that team members should not be expected to inventory their community alone; in order to respond to the needs of their community, they must be informed and equipped with the knowledge of their patient population as determined by the needs of their community from macro scale planning bodies such as the LHIN or Hospice PC Network. At the time of the study, leadership at the Hospice PC Network was in transition and, consequently, there seemed to be a lack of advocacy for teams from individuals beyond the community scale. As a result, a stakeholder argued for more “change champions” at the LHIN scale.

Another stakeholder also cautioned that support for the teams needed to be engrained in a strategy either at the LHIN or provincial scale in order for the teams to overcome their vulnerability at the community scale. There often was an overlap in terms of designating an issue as a LHIN or provincial responsibility:

“… that’s where the strategy comes in so whether there’s a LHIN wide strategy or there’s provincial strategy… but it has to set out some guidelines as to what are the kind of minimum levels of service that are required and then back that up with resources… that are envelope funded, they’re protected because if you put everything in global budgets, things can be taken away in a difficult year, you can turn around and say ‘We’ve got to save ten percent, where are we going to take it from? What’s that little PC team over there – does that match the number we’re looking for?’ It could be as simple as that.” (S2)

Secure funding was one of many resource issues related to the sustainability of teams. In what follows, participants expand on the types of resources from the provincial scale that could help to sustain teams.

#### 3. Province

There is a need to (re) consider funding models at the provincial scale to support collaboration and non-physician team members.

“Because it’s about funding… these teams came about from specialized grant funding… or…, little pockets of money have either continued or died…” (KI 1)

At the time of study, an interest group, the Quality Hospice PC Coalition of Ontario [30] submitted a policy document to the provincial government advocating for a provincial vision, policy, integrated system design and additional investment. The need to support PC programs such as teams working in a shared care model was also included in the document.

An integrated system would enable teams to cross settings of care seamlessly. The teams agreed that the ability for them to access patients in various settings would enable them to provide better coordinated and patient-centred care. For example, only a few of the teams were able to provide consultation in long-term care facilities.

Participants noted that the sustainability of the teams depended on the value that government placed on the home care sector. They pointed out that an infusion of services and funding was not the only solution; there was also a need for clear and concise direction. A provincial strategy would outline clear expectations about service delivery and standardize services so that access to teams was not based on geography alone.

“I think [a provincial strategy would help because]… if it kind of laid out the rules… because you have really [keen] LHINs where they are all cooperating and things are going really well and they get great things, and they get money and they get more things happening and you get other areas that are having some dysfunctions and they can’t get anything, well it’s the same tax payers…” (S2)

While key-informants and stakeholders were able to articulate a system perspective as evidenced above, teams related the provincial role in their sustainability to the provision of ‘on-the-ground’ resources such as human, material and knowledge resources. With additional funding, administrative, psychosocial and bereavement roles, together with an increase in the number of hours for these positions, would be possible.

Additional sources of funding would reduce the vulnerability of the teams. At the time of the study funding for non-physician team members was piecemeal, with a heavy reliance on the support of community partners and in some cases, volunteers. Additional resources would also assist the teams in establishing collaborative partnerships with primary care providers; for example, funding for technological resources, laptops and other electronic devices could help to better coordinate care between team members and primary care providers in a timelier manner.

Overall, additional resources would provide the teams with the ability to provide funders and policy makers with tangible evidence to demonstrate their cost-effectiveness and their ability to keep patients out of hospital.

## Conclusions

The findings of this study add to the limited empirical research on the sustainability of PC programs. The themes generated from the interviews with team members, key-informants and stakeholders were used to conceptualize the factors that contribute to the long-term viability of teams that provide home-based care in a shared care model. Many of the factors discussed below resonate with the sustainability literature, such as the need for resources, policy support and champions eg. [31-33]. A limitation of the study is that it did not include stakeholders from the LHIN and the provincial government. These individuals may have provided additional and/or varied insight.

Sustainability is a commonly used term in health care, often in reference to the system as a whole or a particular program. For instance, the sustainability of a demonstration project is often compromised when its funding period ends and, as a result, it is deemed unsustainable. While the importance of sustainability is understood, the concept remains ill-defined eg. [31,34]. This was illustrated by Hanson et al. [34] through both a review of the literature and interviews with stakeholders about community-based fall prevention programs. Interpretations of sustainability varied among stakeholders within and across three program sites; some considered sustainability as referring to the continuance of the program in its entirety while others related it to certain program components. In their investigation of five primary health care initiatives in Australia, Sibthorpe et al. [32] found that sustainability is influenced by socio-political factors such as, but not limited to: the existence of champions, financial resources, political will, and the capacity of stakeholders. These factors overlapped with the three proposed by Shediac-Rizkallah et al. [31] in their framework for conceptualizing community-based program sustainability. Based on their review of the literature, they surmised sustainability to be affected by: (1) the program layout and how it was implemented; (2) the program setting and/or context, and; (3) the program’s broader external environment. The aforementioned studies focus on primary care and public health; while there is an abundance of literature on the sustainability in these areas, the literature does not adequately address the sustainability of PC service delivery programs. Of the few studies, the majority were not empirical, but rather descriptions of demonstration projects [35], focused on the experiences of physicians eg. [36,37], or considered sustainability entirely from a financial perspective [38].

The inherent difficulties in sustaining PC programs and the lack of guidance addressing these difficulties were recognized by a group of physicians who gathered at an international conference in San Salvatore, Switzerland [37]. Hence, they drew upon their collective experiences to communicate recommendations for developing PC programs and a summary of prerequisite factors that would lay the foundation for an ideal start to a PC program. However, they failed to offer concrete suggestions for established programs. Based on her four-phase model, Kelley [33] elaborates on sustaining a PC program as part of her conceptualization of the process involved in developing PC programs using a community development approach. Once a team is in place (phase three), the fourth phase of the model elaborates the growth of the PC program, which includes a brief description on sustainability. Study respondents, the majority of whom were various health care providers and volunteers on community PC teams, considered additional resources (e.g., both human and material) and policy (e.g., guidelines to formalize the team and roles) as the main contributors to sustainability.

In this study, sustainability was conceptualized from two angles: internal and external. An overview of external sustainability was provided and the merging of data from all participant groups showed that the sustainability of teams was largely dependent on actors and organizations at the local (community), regional (LHIN) and provincial scales.

The three scales are not self-contained or singular entities but rather are connected; Brenner [28] argues that the interrelationships among scales should not be ignored. The decisions at one scale will impact the others. For example, the provincial government recognized the need to improve PC at the community level and an influx of funding in the mid-2000s led to the development of the Hospice PC Networks in each LHIN. The Hospice PC Network in this study sought to improve the quality of care for patients at the community scale while at the same time decrease the use of acute care (which in turn affects both the LHIN and province). While teams attempt to improve the quality of PC care in the community, which is in line with both provincial and LHIN goals, they are seemingly doing so without adequate and dedicated support from the LHIN and provincial government. Returning to the conceptualization introduced earlier, an imbalance in support will compromise the sustainability of teams.

However, in times of fiscal restraint, the provincial government may be positioning itself as a vertical hierarchy making it difficult for community-based practices such as PC teams working in shared care, and champions of this model, to engage in ‘scale jumping’ [29] to influence the LHIN and/or province.While numerous scholars have elaborated the concept of scale theoretically (see [39] for a review), thereby adding to its complexity, few have moved from theoretical to empirical applications of scale in health-related literature. Some have even cautioned against the use of scale as an analytical category eg. [40]. A synthesis of the perspectives and knowledge of the participants helped to inform recommendations for the sustainability of PC teams. For example, at the community scale, teams must continue to engage primary care providers through capacity building. The LHIN, Hospice PC Network and CCAC must align themselves and work in collaboration to champion the service delivery model and to advocate for resources. At the scale of the province, a provincial PC strategy endorsing PC teams and the shared care model would be a step in the right direction. While recommendations specific to each of the three scales of concern are obvious and straightforward, difficulties may result from the fact that scales are inherently relational [25]. Integration and collaboration within and between scales is necessary, as community capacity will inevitably reach its threshold without support of the province, which provides funding to the LHIN. While the community continues to advocate for the teams, in the long-term, they will need additional supports from the LHIN and province. The province has the authority and capacity to engrain its support for teams through a formal strategy.

While these recommendations may seem far-reaching to some, there is a strong impetus for sustaining PC teams. The combination of population aging, increases in chronic disease and preferences for home death will impact community-based care; teams are capable of easing the pressure that this will exert on primary care providers, the acute care sector and the health care system as a whole. In effect, community-based efforts will benefit the LHIN and the province. Taken together, the top priority recommendations demonstrate that it is possible to restore the imbalance between scales if: teams continue to engage primary care providers in capacity building initiatives; the LHIN, Hospice PC Network and CCAC enhance their advocacy efforts; and the province provides financial support.

Another important scale that was absent from the data but may also influence the sustainability of teams, albeit indirectly, is the federal government. Changes at the national scale related to negotiations around transfer payments from the federal government to the provinces for health care, a national home care strategy, as well as knowledge translation activities facilitated through the national interest group, the Canadian Hospice Palliative Care Association (see [21]), have the potential to impact the sustainability of teams.

The sustainability of PC teams that provide home-based care is required to ensure that patients and primary care providers are better supported. PC teams that work in shared care ultimately assist in enhancing the care that is provided to patients and their family members. Without the proper support from community partners, planners and administrators at the larger LHIN and provincial scales, the sustainability of PC teams will be compromised given the limitations of the micro community scale. The people and the organizations at the frontline, at the community scale, will be at the helm of driving change.

The findings highlight the strong willingness of PC teams to provide home-based care in spite of the lack of support. The sustainability of teams working in shared care will require policy leadership and health care transformation in the form of a provincial strategy. The teams are currently working in a health care system that lacks integration between various institutions such as residential hospices, hospitals, and long-term care facilities and the home care sector. Program planners in other jurisdictions may benefit from knowing about the challenges experienced by the PC teams in this study. As program planners turn to innovative home care service delivery models such as PC teams working in shared care to better support primary care providers and patients, it is important to outline the factors that support or hinder the sustainability of teams. The PC teams in this study have demonstrated their value as they address broad health care challenges, including issues related to quality through the provision of holistic and patient-centred care, as well as working with primary care providers in building capacity for end-of-life care. Without additional policy support and/or resources from the provincial government, PC teams will remain vulnerable. The teams may continue community-based efforts but without formalized policy and resources from either the LHIN and/or province, their sustainability will be short-lived. The experiences of the PC teams in this study highlights the challenges to maintaining PC services, which may provide other jurisdictions with insight as to how to better implement and support teams.

## Abbreviations

PC: Palliative care; LHIN: Local health integration network; CCAC: Community care access centre; TM: Team members; KI: Key-informants; S: Stakeholders

## Competing interests

The authors declare that they have no competing interests.

## Authors’ contributions

LD undertook the data collection and analysis. AW oversaw the overall study, cross-checked the data analysis, conceptualized the paper design and illustrations, while providing numerous editorial comments. Both authors read and approved the final manuscript.

## Pre-publication history

The pre-publication history for this paper can be accessed here:

http://www.biomedcentral.com/1472-684X/12/32/prepub
